# Attribution Model of Travel Intention to Internet Celebrity Spots: A Systematic Exploration Based on Psychological Perspective

**DOI:** 10.3389/fpsyg.2021.797482

**Published:** 2021-12-28

**Authors:** Kui Yi, Qingqing Wang, Jun Xu, Bin Liu

**Affiliations:** ^1^School of Economics and Management, East China Jiaotong University, Nanchang, China; ^2^School of Economics and Management, Beijing Jiaotong University, Beijing, China; ^3^School of Statistics, Jiangxi University of Finance and Economics, Nanchang, China; ^4^School of Economics and Management, Jiangxi Science and Technology Normal University, Nanchang, China

**Keywords:** web celebrity attractions, social media influencer, travel attribution, travel intention, empathy process

## Abstract

Previous studies have shown that the empathy process is the main driving factor that triggers tourists’ intention to visit Internet celebrity spots. However, the academic community has not yet formed a unified understanding of the concrete mechanism. Based on this, this study combines the connotations of meme theory and empathy theory and applies Structural Equation Modeling (SEM) to empirically analyze 340 valid samples of Internet celebrity spots visitors to explore the influence mechanism of attributional factors on travel intention. The result shows that mechanism of travel intention can be presented as a psychological model in which travel attribution of tourists to visit Internet celebrity spots is the independent variable, the travel intention is the dependent variable, and the empathy process is the intermediary variable. The influence intensity of internal attribution on affective empathy is higher than that of external attribution, while the influence intensity of external attribution on cognitive empathy has a comparative advantage, and there is a significant difference between them. Empathy process has a significant mediating effect on the relationship between travel attribution and travel intention of tourists to visit Internet celebrity spots, and the mediating effect of affective empathy is significantly greater than that of cognitive empathy. Overall, this study reveals the segmentation elements with strong explanatory power in the behavior of “internet celebrity spots punch in,” examines the practical effect of empathy process in the behavioral intention of traveling, and provides a theoretical reference for the transformation and upgrading of tourist destinations and marketing planning of online communication in the future.

## Introduction

The iteration and popularity of Internet technology is penetrating people’s lives in all aspects (Zhou and Whitla, 2013), and the means of communication between people is undergoing disruptive changes (Wang et al., 2021; Yang et al., 2021a). Facebook, Twitter, Instagram, and other social media have spawned a number of industries based on Internet carriers. In this context, Internet celebrity economy emerged. As a new thing, the current Internet celebrity economy is developing rapidly with its strong social asset realization ability (Sun et al., 2021), user-generated content (UGC) dissemination benefits, and quasi-social interaction characteristics favored by capital markets in various industries (Suwan, 2018), and its deep laws have triggered many thoughts in the tourism industry. Specifically, the occurrence of Internet celebrity economy implies the rise of visual media and the further widening of interaction channels for the public. Tourism followers can form a deep knowledge of tourism attraction through the reading and discussion of content information on social platforms, and they can use the forwarding function to realize the effective transformation from audience to communicator of tourism destination information dissemination. Since the act of reading, discussing, and retweeting depends entirely on the immediate thoughts of the tourism followers, audience, and communicator identities do not exist in a fixed way, which means tourism followers can change freely between audiences and communicators (Albuquerque et al., 2012). Thus, it can be seen that the tourism model derived from Internet celebrity economy is an innovative form of tourism where followers participate in the operation of intellectual property centered on visual elements (Geng et al., 2020), which leads to a word-of-mouth effect in the visual meaning generation and communication path. It can definitely gather a lot of social attention in the short term, attract groups of tourists, and generates economic effects (Judith and Dina, 2006). Such patterns build a link between tourism destinations and social media, which can attract tourism followers faster and easier. Because the process of this model is similar to the “punch in and punch out” mode of enterprises, the tourism model derived from the Internet celebrity economy is often called Internet celebrity spots punch in model. The emergence of Internet celebrity spots punch in model indicates that the marketing strategy of tourist destinations is undergoing a transformation, which means that the influential environment formed by tourists is changing dramatically the direction of tourism industry development. Existing research has systematically explored the formation and evolution of “Internet celebrity spots punch in” behavior (Su, 2020). Value and significance of “Internet celebrity spots punch in” model construction (Pan, 2021) and the forms and paths of its marketing have been systematically explored (Gao, 2019).

From a sociological perspective, the concept of “Internet celebrity spots punch in” is often considered by academics as a kind of visitation behavior transformed from online attraction by users who are emotionally influenced by individuals and communities producing information related to the Internet. Specifically, this process often begins with the information publisher’s sharing of their own travel activities and experiences, as well as marking the geographic location and time traveled. In general, this is the act of providing the communication audience with effective information about travel behavior preferences and patterns (Girardin et al., 2008). With the continued transmission of the psychology of community recognition, the phenomenon of “Internet celebrity spots punch in” is given a cyclical attribute, cycling back and forth, until the fervor of the destination weakens as the attention and novelty fades out of the center of online discussion (Zhao et al., 2021). It can be seen that the formation and evolution of “Internet celebrity spots punch in” behavior always revolves around the requirements of tourism destination image construction, which is the product of public emotional attachment. It is different from the construction of the tourism destination image in traditional official media that is more attached to the transformation of social power relations by the network ecology, and to some extent breaks the evaluation mechanism based on brand reputation (Milner, 2010). Instead, it takes the degree of attention and buzz as the core of tourism destination level evaluation criteria (Xia et al., 2017; Fu, 2021), or the growing number of clicks, likes, and retweets as evaluation indicators, or the size of fan following groups and the frequency of interactive discussion characteristics of online communities as observed variables (Huang et al., 2019). A large number of studies on the construction of evaluation systems point out the formation of “Internet celebrity spots punch in” as the generation of a typical word-of-mouth communication business model in the age of attention economy. Following the popular hotspots, focusing on IP boom, exploring the “highly recognizable and differentiated” mental symbols for segmentation and positioning (Song et al., 2021), supplemented by the catalyst of big data technology empowerment, “Internet celebrity spots punch in” has been in the process of continuously optimizing its path. Meanwhile, the “Internet celebrity spots punch in” has also created various image representations of tourist destinations. Under the background of the proliferation of Internet users, the “Internet celebrity spots punch in” model is becoming more and more attractive to modern people, so whether it is online marketing or offline operation, it is one of the most effective marketing paths for tourism destinations.

As a flourishing cultural phenomenon, emergence of “Internet celebrity spots punch in” is in line with the evolutionary law of human needs and the desire to satisfy capital, and it will continue to be a hot issue in society for a long time. Despite research on the formation and evolution of “Internet celebrity spots punch in” sharing some valuable results, there are few studies in current literature that are combined with a psychological perspective. “Internet celebrity spots punch in” is actually the product of the occurrence and evolution of psychological resonance and emotional resonance; therefore, it is necessary to conduct the research and study to explore its attribution. To this end, this study will address the following questions: First, what are the potential and observed variables that contribute to generating the travel intention of Internet celebrity spots, considering both communication psychology and travel behavior? Second, should individual differences in empathy be considered as a factor for travel intention of “Internet celebrity spots punch in,” namely, should the influence of each attribution factor on travel intention to Internet celebrity spots be mediated by the empathy process? Third, if empathy can act on the generation of travel intention of “Internet celebrity spots punch in,” is the rational element of cognitive empathy more influential or the emotional element of affective empathy more influential? These three research questions will systematically interpret the generation mechanism of travel intention of “Internet celebrity spots punch in.” On the whole, the behavior of setting Internet celebrity spots as tourist destinations or “Internet celebrity spots punch in” usually engages in social interaction on the Internet, which is a new social mode for people to break the boundaries of physical space. The research on this can provide theoretical reference for the sustainable development of tourist destinations in the new era.

## Theoretical Foundation and Hypothesis Development

### Theoretical Foundation

#### Meme Theory

Meme theory is an important concept to understand the behavior of people mimicking Internet celebrity to visit Internet celebrity spots. It is similar to the continuous self-replication of biological genes in the process of evolution, and the meme is a re-replication process in the process of cultural development. Since its introduction in 1976, the research and development of meme theory mainly includes two stages: biological metaphor stage (1970–2000) and cultural theory stage (2000–present). The former focuses on the inferential analysis of memes and genes, similar to biological transmission such as virus transmission, so as to explore specific sociological laws such as replication, transmission, and variation of memes, which enrich and improve the theory of memes. The latter is the reflection of existing problems in external fields such as culture and psychology. As meme theory is presented as a multidisciplinary cross-application, the application of meme theory has formed a certain accumulation of experience and cognitive consensus in humanities and social sciences, psychology, and online marketing (Davi, 2007; Nigel et al., 2014; Spitzberg, 2014), and academic circles believe that memes reproduce by self-replication and transmission of content and form, and unpredictable cultural variations occur in the process.

That meme of “Internet celebrity spots punch in” is a cultural phenomenon that spreads through replication and mutation, and any destination travel activity can become a meme (Jolliffe and Farrington, 2006). Thus, it can be considered as a unit of popular culture transmitted, imitated, and transformed by Internet visitors and as a cultural experience created and shared by Internet visitors (Shifman, 2013). It is the infectious images, videos, and buzzwords that spread rapidly in a mimetic environment with constant imitation and modification by users (Coleman, 2012). Evidence shows that the meme in the “Internet celebrity spots punch in” has been characterized by significant variation from the beginning to the end, and its effect goes far beyond “self-replication.” Massive and atomized Internet tourists’ active participation is an important sign of the formation of memes. Any Internet user can become an anonymous creator of a meme (Knobel and Lankshear, 2007). In a word, the tourism field has always been strongly interested in the exploration of information product memes not dominated by “use value” and the consumption intention and cultural tendency reflected behind them. These achievements and theories have laid a solid foundation for this research.

#### Empathy Theory

The term *empathy*, first coined by the British psychologist Edward Titchener in 1909, means “to feel” (Chen, 2018), which is to recognize and understand an individual’s psychological feelings through affective empathy, emphasizing being motivated (Sinclair et al., 2017). Implementation of the behavior of “Internet celebrity spots punch in” is derived from the empathy of tourists in the interactive ritual chain, which is not only a psychological state but also represents a cognitive ability. Through the perspective of empathy theory, it is possible to effectively analyze the psychological phenomenon of interpersonal interaction of Internet users, which is a social psychological process with both dynamic and directional characteristics (Brown et al., 2019). Empathy communication is a communication theory based on empathy, which interprets the psychological characteristics and emotional changes of tourists after obtaining destination information. It is an effective perspective to analyze the psychological and emotional resonance characteristics of tourists visiting Internet celebrity spots (Katrina et al., 2019). Therefore, to clarify how “Internet celebrity spots punch in” resonate with tourists and promote tourists to have tourist behavior intention have great significance to the development of tourism economy.

In addition to being a perspective, the structure of empathy is mostly used as a measurement tool in empirical studies. So far, commonly used empathy scales include the negative emotionality scale, the empathy quotient scale, the basic empathy scale, and the interpersonal reactivity index scale (Baron-Cohen and Wheelwright, 2004; Jolliffe and Farrington, 2006; Vachon and Lynam, 2016). Among them, two of these measures, cognitive empathy and affective empathy, have reached a consensus. Highly empathic individuals understand the feelings of others, called “cognitive empathy,” which is also empathic accuracy in the traditional sense, including the ability to detect and understand emotional expressions (Ickes, 1993). The further indirect experience of others’ emotions, called “affective empathy,” involves the visitor’s emotional response which is consistent with the target in terms of validity (Batson, 2009). The IRI scale was selected as the measurement scale for punch card empathy communication, by considering that most of tourists’ travel motivations in this study are positive emotions, which is matched with its strong interdisciplinary applicability.

To sum up, according to meme theory, anchor is the overall goal of travel intention attribution model analysis of tourists who punch in Internet celebrity spots, systematically exploring the psychological characteristics of “Internet celebrity spots punch in” behavior, exploring the intuitive manifestation of internal and external attribution through the rational behavior theory, and finally establishing the specific composition of the empathy process analysis perspective.

Based on this, the study will discuss the relationships between travel attributions and empathy processes, empathy processes and travel intentions, and travel attributions and travel intentions of Internet celebrity spots tourists.

### Hypothesis Development

#### Travel Attribution and Travel Intention

As early as the 1960s, psychologists put forward two types of attribution, namely, internal attribution and external attribution. The former is based on the internal personal factors of the actor, and the latter is based on the external environmental factors of the actor (Heider, 1958). Kelley (1967) further explained attribution from the perspectives of actor, objective stimulus, and situation. Compared with Heider’s attribution, the actor in Kelly’s attribution still belongs to Heider’s internal attribution, while objective stimulus and situation belong to external attribution (Kelley, 1973).

Therefore, in this study, internal attribution can be interpreted as the psychological needs and internal motivations that drive tourism consumers to produce certain behavioral tendencies (Baumeister, 1982), including group identity, self-cognition, and self-presentation (Baumeister, 1982). According to Maslow’s hierarchy of needs theory, when the low-level physiological needs and security needs have been sufficiently secured, people will start to seek higher-level psychological needs such as social needs, respect needs, and self-actualization needs. These demand drives can be effectively satisfied in the behavior of “Internet celebrity spots punch in” and affect their behavioral willingness, which has been recognized by the academic community (Cui et al., 2021). Specifically, through “Internet celebrity spots punch in” a virtual-reality linkage model formed, which provides the possibility for tourism-intending people to achieve the goals of playful entertainment and social interaction (Zhao et al., 2021). Through the group identification of tourist destinations, information sharing and resource exchange can be promoted, and users’ intention to visit can be triggered through the formation and maintenance of user content generation, forming a new way of resource allocation in the new era (Chen et al., 2015; Jana et al., 2021). Besides, active self-presentation and passive self-cognition among groups are important expressions of psychological needs, which are conducive to the generation of intentions and the cultivation of positive emotions, and then stimulate corresponding behaviors (Giamo et al., 2012). In short, participation groups of “Internet celebrity spots punch in” have consistent psychological needs in the process of interaction, and there is a positive internal logical relationship between the travel intention and internal attribution (Li and Zhang, 2021; Meng et al., 2021), that is, the group identification, self-presentation, and self-cognition contained in internal attribution can make the tourist group have a more positive intention to travel to a certain scenic spot. A large number of practical experiences shows that with the rapid development of big data, potential tourists can obtain all kinds of tourism information through more diversified channels, and then form an original image and get the scenic spots worth going to. Through comprehensive analysis of gradually formed image characteristics, potential tourists evaluate the value of tourism activities in the destination and choose the best solution to meet their needs.

On the other hand, external attribution can be interpreted as the influence of external environmental factors such as destination image information and subjective norms that tourists are exposed to. The former refers to the content and form of meme communication formed under the new tourism mode of “Internet celebrity spots punch in.” It is known from existing studies that various types of tourism information about a destination affect the tourist’s perception of the destination’s image, and that this perception is constantly built and modified, ultimately affecting the tourist’s decision-making behavior tendencies (Fakeye and Crompton, 1991). After potential tourists choose the best solution, the tourism destination experience activity begins. At this time, potential tourists who have become real tourists will re-understand the image of the tourist destination according to what they see and hear, and then become a new information spreader and exert impact on the travel choices of other potential tourists (Awaritefe, 2004). In addition, according to the planned behavior theory mentioned above, the subjective norm is defined as the social pressure that tourists perceive when they participate in the tour of Internet celebrity spots, which reflects the pressure or influence given by important people or organizations around them (Armitage and Conner, 2001). When tourists realize that important people or organizations around them support their participation in the tourism activity, tourists are likely to participate in the tour of Internet celebrity spot, so as to adapt themselves to the expectations and needs of the surrounding groups and the needs of social development (Han and Kim, 2010). That is, the stronger the support of important people or organizations, the stronger the willingness of tourists to participate in travels to Internet celebrity spots (Harrison, 1995), which shows that external attribution can make the tourist group have a more positive willingness to visit Internet celebrity spots. In other words, external attribution has a significant impact on their travel intention. In summary, this paper proposes the following hypothesis.

H1a: Internal attribution is positively related to travel intention.
H1b: External attribution is positively related to travel intention.

#### Travel Attribution and Empathy Process

In essence, to explore the psychological laws behind “Internet celebrity spots punch in,” it can be regarded as a kind of ritual consumption, that is, to let a moment be given a special spiritual connotation, tourists hope to satisfy their intrinsic psychological needs through photo rituals, and this demand can be specifically divided into three categories. The first is identity. Identity is reflected in the visitor’s desire to be part of a group for a long time (Biddle, 1991) and the desire for interpersonal relationships in the virtual world (Sander and Putnam, 2010). This non-product-oriented consumption is a behavior that arises to cater to a certain group of people and often reflected in the need to satisfy one’s sense of identity (Cohan, 2001). In fact, the phenomenon stems from a group effect (Spry et al., 2011), which can change visitors’ attitudes and attention to buying a product or service (Choi and Rifon, 2012), allowing group reputation to be transmitted to a specific product or service through media communication (Thomson, 2006), where the group includes its own social circle or fans who like stars or popular activities on video software. The second is self-presentation. The self-presentation of “Internet celebrity spots punch in” behavior implies that individuals attempt to influence others’ impression management of themselves. It is generally manifested as an active presentation by tourists after a tour with the aim of establishing a specific, positive homogeneous behavior in the minds of others (Lee et al., 1999), completing the self-labeling process and seeking community or social approval of the ideal self-image (Arkin et al., 1980). In other words, in the social media “circle,” tourists often try to meet the feedback needs of the “stage audience” and then reconstruct their own status to achieve the social value and meaning of the destination visit. The third is self-awareness. The emergence of socialization has given rise to new human needs, and the emergence of scenario-based social consumption has further reinforced such preferences. Specifically, tourists display their travel routines in social platforms as a way to engage in social interactions with others (Perez et al., 2018; Ogaard et al., 2019). In recent years, the evolution of the consumer revolution has realized the high-speed transmission and coverage of information, and scene marketing is gradually taking shape. Under the guidance of the globalization of network information, the two-way empathy mechanism between tourists’ psychology and communication interaction has become a key issue of concern in tourism academia. Regarding cognitive empathy, scholars are divided, presenting the following three main views.

First, psychologist Batson (2009) distinguishes empathy as a phenomenon that includes understanding others’ hearts, imitating actions, putting oneself in the shoes of others in an aesthetic sense, and feeling the feelings of others. The second is the “three-component theory,” which suggests that empathy can be divided into three components: “emotion infection,” “perspective selection,” and “empathic attention” (Jia and Chen, 2020). The third is the “dual process theory,” which divides the empathy process into two processes: affective empathy and cognitive empathy, arguing that empathy is manifested from innate emotion infection and emotion recognition on the one hand, and from experience-generated emotional understanding on the other (Galinsky et al., 2006). The three types of perspectives, respectively, explain the phenomenon, components, and process of empathy, which can jointly build the core points of empathy communication concept in “Internet celebrity spots punch in.” In the context of the Internet era, the rational cognition and emotional cognition of known users are coexisting and can continuously improve themselves in the evolutionary sequence of information transmission.

Thus, no matter if it is self-presentation, self-perception, or group identity, the relational need for internal attribution motivates tourists to perceive the psychological perception of interpersonal presence and satisfaction expressed by “Internet celebrity spots punch in” behavior, and to expect the same emotional feedback through “frame of reference” behavioral replication and personality variation (Benaim, 2018), that is, affective empathy and cognitive empathy.

Therefore, this study proposes the following hypothesis.

H2a: Internal attribution is positively related to affective empathy.
H2b: Internal attribution is positively related to cognitive empathy.

On the other hand, external attribution can be interpreted from two aspects, namely, the expression form of destination image in Internet immersion communication in the theory of reasoned action model and subjective norms. The former includes the content and mode of Internet celebrity meme communication. The latter is based on the operational definition of subjective norms in theory of reasoned action and contains three dimensions of social influence, interpersonal influence, and self-control (Bartle and Harvey, 2017). The empathy process is a kind of emotional and psychological feedback of tourists to interpersonal construction. Interpersonal interaction will cause tourists to have a series of psychological complex emotional processes, while the meme content is the communication carrier of interpersonal interaction, and the subjective norm is the external environment influencing factor of the communication carrier. Both of them can change tourists’ tourism psychology (Boyd and Richerson, 2000). With the rapid development of the Internet era, faster and wider dissemination of tourism destination image is no longer out of reach, and the real-time sharing effectiveness provided by online platforms can make people know and understand the expected or enthusiastic major “online attractions” in a short period of time (Lam et al., 2021). Specifically, the tourism destination image belongs to the subjective category, which is the impression and expectation formed by a person’s subjective perception of a specific destination, as well as emotional perception. Tourism destination image has a close relationship with tourists’ or potential tourists’ behavioral motivations, tourism decisions, perceptions of service quality, and satisfaction (Asnawi, 2021), and it can influence potential tourists’ decision-making behavior and meme perception levels (Wu and Liang, 2021). Drawing on the expected value theory of behavioral motivation, meme content may be significantly related to emotional perceptions of tourist attractions, and the better the tourist’s image of Internet celebrity spots they punched in, the greater its empathic perceptual validity (Gibson, 2010). Many studies have found that in most contexts, destination images may not be directly related to one’s own travel memories and planning, and people are driven by curiosity or desire to know and show strong attention to the Internet celebrity spots. The audience will also gather and learn about the destination image through various channels to satisfy their curiosity and novelty (Wu and Liang, 2021). At the same time, due to the periodicity of the hot image of Internet celebrity spots, the uncertainty of its permanence is particularly prominent. After receiving the destination image, potential tourists tend to focus their attention on hot spots for a period of time. They actively encourage themselves to continue to accept media information, so as to deepen their understanding (Liang and Xue, 2021). Conversely, affective empathy will also lead the media audience to actively focus on and participate in the user-generated content (UGC) process.

With the strengthening of the audience’s understanding and cognition of the destination, their enthusiasm to participate in the dissemination process of the meme is gradually rising. Tourists will try to show their value and influence in the process of Internet celebrities’ punching in through their own efforts, so as to deepen their empathy. Travelers will try to show their value and influence in the “Internet celebrity spots punch in” process through their own efforts, thus deepening the empathy performance (Zhuang et al., 2021). The interactive nature of the Internet, the integration of multimedia, and the anonymity of opinion expression are all characteristics of the information carrier formed by “Internet celebrity spots punch in” (Cheunkamon et al., 2020), which together prompt audiences to choose popular tourist destinations based on their own perceptions, emotions, and attitudes. Among them, collective discourse and group gaze in the context of subjective norms can further consolidate and reinforce individual cognitive and behavioral choices in behavioral intentions while creating emotional contagion in the audience (Han et al., 2020). In other words, both the theory of reasoned action and theory of planned behavior believe that subjective norms have a direct effect on tourists’ emotional perceptions, and individual behaviors and trends are usually affected by the surrounding environment, especially by the views and opinions of people who are closely related to them and have important interests (Joo et al., 2020). For people with a strong dependency mentality, the advice of others will even directly influence the final decision. Travel is usually done as a group activity, and most tourists rarely travel alone, which will be more influenced by the group and social environment, showing a certain sense of regularity (Bo et al., 2020).

In summary, from the perspective of the empathy process, the more visitors can positively perceive the meaning of subjective norms and travels to Internet celebrity spots, the more negative affective hindrances can be eliminated. When the content of “Internet celebrity spots punch in” meme exceeds the boundary of tourists’ psychological equilibrium, it leads to empathic fluctuations in tourists’ psychology. Among them, the mood fluctuations and psychological changes triggered by the destination image invoked by the meme content are synthesized as the affective empathy and cognitive empathy of tourists (Wu et al., 2021). Therefore, the following hypothesis is proposed in this study.

H2c: External attribution is positively related to affective empathy.
H2d: External attribution is positively related to cognitive empathy.

#### Empathy Process and Travel Intention

With the widespread use of psychological discipline concepts in tourism, empathic psychology has gradually become an important perspective in characterizing tourism behavior and a key factor in observing certain attitudes and behavioral pre-test variables of tourists (Nugroho et al., 2021). It has been empirically shown that empathy is an important component in generating identity and attachment, which helps to understand others’ perspectives, needs, and intentions and is crucial in dissecting tourist services and willingness to visit (Pizam, 2015; Wandebori, 2017). Empathic psychology means that feeling the cognitive and emotional experiences of others can generate both cognitive and emotional responses and can effectively communicate information to others. It can be seen that empathic psychology can also be understood as a learned and trained skill. Driven by the capital of communication technology, a specific online atmosphere induces travel intention among Internet users to visit Internet celebrity spots. It is known from empathic communication theory that communication messages can cause psychological empathy and emotional resonance among audiences. Under this effect, travel sharing messages generated by online communicators usually show two aspects of empathic performance (Bauman et al., 2020). On the one hand, it is manifested as emotional infection, and on the other hand, it is manifested as conscious emotional sharing based on emotional infection (Sharifi-Tehrani et al., 2019). Specifically, the most common characteristics of emotional infection in destination attraction communication are the sense of participation and the sense of concern, that is, the needed sense of presence of network media generated by the behavior of receiving feedback, thumbs up, and replies from online tourism audiences (Zamanillo et al., 2019). The bottom-up emotional sharing process of empathy communication is of great value for the further dissemination of destination marketing. The tourism effect of “Internet celebrity spots punch in” will trigger social repercussions, which will actively stimulate tourists to generate corresponding symbolic consumption psychology and behavioral feedback of “template nesting.” The sharing of emotions can help tourists create a sense of achievement and gain in their travel records (Émilie, 2017).

The emotional resonance process of empathic communication is generally based on a bottom-up stimulation process. The empathy process itself is not unlimited, which needs to be put into practice. As a theoretical framework for understanding the influences on tourists’ intentions, the theory of planned behavior asserts that behavior is determined by behavioral intentions (de Kervenoael et al., 2020). Expectancy value theory, on the other hand, suggests that behavioral intentions depend on the individual’s evaluation of the expected outcome and the expectation that the behavior will lead to the outcome, so the overall shared emotional knowledge of the visitor is key to understanding customer behavior (Velvet, 2020). On the premise that satisfaction is considered as behavioral intention, community perception indirectly influences revisit intention through the mediating effect of satisfaction on travel experience (Jang and Chung, 2019). Another part of the study concluded that the empathic perception of the destination directly and positively influences the travel intention of tourists, that is, the higher the sense of empathic experience of tourists, the stronger their travel intention (Laing and Frost, 2018).

Therefore, this study proposes the following hypothesis.

H3a: Cognitive empathy is positively related to travel intention.
H3b: Affective empathy is positively related to travel intention.

#### Mediating Role of Empathy Process

Many researchers show that individual self-construction such as self-presentation and self-perception cannot be separated from the influence of group identity, which is the result of self-construction. In the era of social media, subjects neglected by traditional media can strengthen individual self-awareness, realize “self-construction,” and enhance group identity through the travel meme carrier of social media Internet celebrities and normative power of group communication. These are key factors in generating emotional perception and empathy mapping (Iina et al., 2018). However, the above research results only show that the psychological demand generated by tourist groups of Internet celebrity spots and the passive external influence are important prerequisites for driving tourism consumption. In addition, researchers have recognized empathic psychology as an important factor in enhancing individuals’ willingness to act, but a broader consensus has not been reached (Nugroho et al., 2021). Since the beginning of the formation of travel intention to Internet celebrity spots, it is more important to produce the stimulus response of psychological demand, which is realizing the missing psychological factors of the current situation, and then produce unbalanced cognition and seek adjustment and recovery (Velvet, 2020). Effective psychological empathy enables the Internet celebrity tourism group to stand in the perspective of the Internet celebrity tourism information publisher and grasp the inner world of the disseminator. Effective psychological empathy enables travel groups of “Internet celebrity spots punch in” to stand in the perspective of the publisher who releases travel information of Internet celebrity spots to grasp the inner world of the communicator, and to reduce the time and money costs of group behavior formation, which requires attention to the mechanism of the psychological transition variable of the empathy process. At the same time, some scholars believe that there exist some factors that often do not directly promote the formation of travel intention to Internet celebrity spots and guarantee the transformation of behavioral willingness, but through certain mediating factors to stimulate their travel motivation and mobilize the already formed travel needs of tourists or potential tourists to recognize and choose that online tourist destination. And in this, the empathy process is the mediating mechanism in the process of driving behavioral choices, which is the mediating factor between travel attribution and travel intention (Crossley, 2017).

Therefore, this study proposes the following hypothesis.

H4a: Cognitive empathy mediates between internal attributions and travel intention.
H4b: Affective empathy mediates between internal attributions and travel intention.
H4c: Cognitive empathy mediates between external attributions and travel intention.
H4d: Affective empathy mediates between external attributions and travel intention.

**FIGURE 1:**
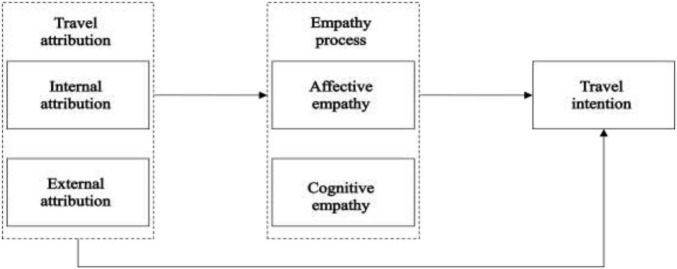
Theoretical model.

In summary, the following research model can be formed according to research hypotheses (as shown in Figure 1).

## Materials and Methods

### Participants and Procedure

In this study, “China Xi ‘an wrestling bowl wine” and “Hongyadong in Chongqing” were selected as the subjects, both of which are typical tourist spots of China’s “famous net roots” and are on the list of “must-see net roots tourist places” in China; therefore, the research conducted on them is representative. To further ensure the rationality and validity of the questionnaire design, a small range of online pre-survey was conducted for users who participated in the punching video before the formal survey. The research results showed that the scale design was well-structured and met the requirements of reliability and validity test.

On the basis of the preliminary survey, the official research questionnaire was collected from the publishers and commenters of “wrestling bowl wine” and “Hongyadong” videos on TikTok, Bilibili, and other mobile video sites. A total of 340 questionnaires were collected from the publishers and 120 questionnaires were from commenters. Based on these two parts of data, the summaries of 460 preliminary questionnaires were gathered. Further, we deleted invalid questionnaires that took <1 min to fill in and scored the same items. Finally, 340 valid sample data were obtained, with an effective rate of 73.91%.

As can be seen from the basic characteristic information of the surveyed tourists (as shown in Table 1), in terms of gender ratio, the surveyed tourists include 182 females and 158 males, accounting for 53.5 and 46.5% of the total, respectively, with a harmonious gender ratio. In the aspect of age structure, the majority are teenagers under 30 years old, accounting for 79.2% of the total number. With education level distribution, the majority of the interviewed tourists have a university degree, accounting for 67.5% of the total number. In terms of education distribution, most respondents have a bachelor’s degree, 228 people, accounting for 67.1% of the total, which means they have the ability to fill in the questionnaire. In respect to consumption structure, 154 respondents, accounting for 45.3% of the total, are in the range of RMB 2,001–5,000.

**TABLE 1:** Descriptive statistical analysis.

Variables	Item	Frequency	%	Variables	Item	Frequency	%
Age	Under 24 years old	161	47.4%	Gender	Male	158	46.5%
	25–30 years old	108	31.8%		Female	182	53.5%
	31–40 years old	67	19.7%	Educational background	High School and below	10	2.9%
	41 years old or above	4	1.2%		Specialized	11	3.2%
Consumption level	Below 2,000 RMB	90	26.5%		Bachelor’s degree	228	67.1%
	2,001–5,000 RMB	154	45.3%		Postgraduate and above	91	26.8%
	5,001–10,000 RMB	34	10.0%	Profession	Students	136	40.0%
	10,000 RMB or more	62	18.2%		Employees of state-owned enterprises	94	27.6%
Travel experience to Instagram-worthy location	Yes	200	58.8%		Civil servants	65	19.1%
					Freelancer	19	5.6%
	No	140	41.2%		Freelancer	26	7.6%

### Measure

Specifically, the SEM method is adopted to verify the hypotheses as discussed above by empirical analysis. The Likert 5-point scale was used to measure all measured variables, with 1 indicating *strong disagreement* and 5 indicating *strong agreement*.

#### Internal Attribution

The attribution of travel intention to Internet celebrity spots is divided into internal attribution and external attribution, among which the internal attribution is divided into “self-presentation,” “group identification,” and “self-cognition.” This is based on the connotation definition and operational description of internal attribution of behavior by Tajfel (1986) and Bernard (2010). The typical measurement item in the scale was, “I will carefully select the pictures of caging before uploading them to social media after playing,” and the Alpha of the scale was 0.853.

#### External Attribution

The external attribution scale of Fishbein and Ajzen (1975) and Armitage and Conner (2001) was used in this paper, and “meme content” and “subjective norms” were selected for this study, and the alpha of this scale was 0.886.

#### Cognitive Empathy and Affective Empathy

The affective and cognitive measure of empathy scale by Vachon and Lynam (2016) was used in this paper, with an alpha of 0.890 for the cognitive empathy scale and 0.881 for the affective empathy scale.

Travel intention, which is divided into three dimensions: “willingness to play,” “willingness to recommend,” and “willingness to share,” was measured using Cronin and Taylor’s (1992) definition and elaboration of the dimensions of behavioral intention were used to measure travel intention, and the alpha of this scale is 0.807.

These items above constitute the initial measurement scale, and AMOS 26.0 software was used to analyze collected data to deal with the relationship and validation between a series of variables, mainly including confirmatory factor analysis, discriminant validity, model fit, and path coefficients.

## Research Results

After six iterations of rotation, the rotation component matrix is obtained by using maximum variance orthogonal method (as shown in Table 2). Through factor analysis, it can be seen from the rotated component matrix that the first factor has a high load value on six variables IA1–IA6, the second factor has a high load value on six variables EA7–EA12, the third factor has a high load value on four variables CE1–CE4, and the fourth factor has a high load value on four variables AE5–AE8. Further combining with the questionnaire index system and the research hypothesis model, it is concluded that factor 1 is internal attribution, factor 2 is external attribution, factor 3 is cognitive empathy, and factor 4 is affective empathy.

**TABLE 2:** The rotation factor load.

Factor	Item	Load	Factor	Item	Load
Factor 1	IA1	0.71	Factor 2	EA7	0.78
	IA2	0.73		EA8	0.76
	IA3	0.72		EA9	0.73
	IA4	0.64		EA10	0.67
	IA5	0.69		EA11	0.53
	IA6	0.50		EA12	0.58
Factor 3	CE1	0.83	Factor 4	AE5	0.59
	CE2	0.74		AE6	0.52
	CE3	0.67		AE7	0.70
	CE4	0.61		AE8	0.76

*IA indicates sustained internal attribution, EA indicates external attribution, CE indicates cognitive empathy, AE indicates affective empathy.*

### Confirmatory Factor Analysis

The results of the Confirmatory Factor Analysis (CFA) study are shown in Table 3. The standardized factor loadings of all dimensions are between 0.641 and 0.870, and the Average Variance Extracted (AVE) for all variables ranged from 0.515 to 0.617, which is greater than the criterion of 0.5, and the Composite reliability ranged from 0.807 to 0.858, both greater than the criterion of 0.7. Therefore, the results of the validation factor analysis in this study all met the criteria, the convergent validity was good, and all the constructs had good convergent validity.

**TABLE 3:** Confirmatory factor analysis.

Construct	Item	Significant test of parameter estimation				Item reliability		Convergence validity	Composite reliability
		*Unstd*	*S.E*	*z*-value	*p*	*STD.*	*SMC*	CR	AVE
Internal attribution (IA)	IA 1	1.083	0.119	9.101	0.000	0.672	0.579	0.829	0.604
	IA 2	1.282	0.138	9.290	0.000	0.787	0.591		
	IA 3	1.185	0.119	9.958	0.000	0.755	0.549		
	IA 4	1.034	0.119	8.689	0.000	0.712	0.469		
	IA 5	1.229	0.127	9.677	0.000	0.852	0.464		
	IA 6	1				0.870	0.295		
External attribution (EA)	EA1	0.995	0.095	10.474	0.000	0.796	0.399	0.837	0.565
	EA2	1.123	0.086	13.058	0.000	0.715	0.630		
	EA3	1.179	0.092	12.815	0.000	0.760	0.614		
	EA4	1.156	0.083	13.928	0.000	0.685	0.729		
	EA5	0.966	0.059	16.373	0.000	0.737	0.439		
	EA6	1				0.729	0.481		
Cognitive empathy (CE)	CE1	1				0.684	0.553	0.807	0.515
	CE2	0.831	0.071	11.704	0.000	0.641	0.426		
	CE3	0.953	0.093	10.247	0.000	0.756	0.401		
	CE4	0.847	0.086	9.849	0.000	0.689	0.381		
Affective empathy (AE)	AE1	1.089	0.125	8.712	0.000	0.654	0.370	0.841	0.522
	AE2	1.365	0.15	9.100	0.000	0.764	0.435		
	AE3	0.997	0.099	10.071	0.000	0.701	0.259		
	AE4	1				0.743	0.229		
Travel intention (TI)	TI1	1				0.773	0.282	0.858	0.617
	TI2	0.836	0.098	8.531	0.000	0.756	0.194		
	TI3	1.068	0.117	9.128	0.000	0.838	0.243		
	TI4	1.046	0.108	9.685	0.000	0.763	0.287		
	TI5	0.975	0.091	10.714	0.000	0.814	0.414		
	TI6	1.182	0.113	10.460	0.000	0.765	0.381		

*STD, Standardized factor loadings; SMC, Square Multiple Correlations; CR, Composite Reliability; AVE, Average Variance Extracted.*

*IA indicates sustained internal attribution, EA indicates external attribution, CE indicates cognitive empathy, AE indicates affective empathy.*

### Discriminant Validity

Table 4 reports the discriminant validity for the measurement model, and the square roots of the AVE are reproduced on the diagonal. Discriminant validity is the extent to which the measure is not a reflection of some other variables. It is indicated by low correlations between the measure of interest and the measures of other constructs. We have examined discriminant validity using Fornell and Larcker’s (1981) recommendation that the square root of the AVE for each construct should be higher than the correlations between it and all other constructs. Table 4 shows that the squared root of AVE for each construct is greater than the correlations between the constructs and all other constructs. As shown in Table 4, our results support Fornell and Larcker’s (1981) requirement of discriminant validity.

**TABLE 4:** Discriminant validity for the measurement model.

Variables	Mean	SD	AVE	1	2	3	4	5
1. Internal attribution	3.8652	0.5907	0.604	**0.777**				
2. External attribution	3.9265	0.5891	0.565	0.683	**0.752**			
3. Cognitive empathy	3.8838	0.5432	0.515	0.569	0.508	**0.718**		
4. Affective empathy	4.1243	0.5570	0.522	0.601	0.634	0.529	**0.722**	
5. Travel intention	3.8431	0.5229	0.617	0.776	0.724	0.588	0.692	**0.785**

*The items on the diagonal on bold represent the square roots of the AVE. Off-diagonal elements are the correlation estimates.*

### Model Fit Degree

In this study, the model fit degree index refers to the model of model fitness analysis, and the 9 most extensive fitness indicators are used for analysis (Jackson et al., 2009). Since the SEM sample is larger than 200, it is easy to cause the chi-square value to be too large and lead to poor fit, so the fit value needs to be corrected by Bootstrap (Bollen and Stine, 1992). After passing the Bollen-Stine Bootstrap correction model, all the fitness indicators in this study have been passed (as shown in Table 5), more precisely, in particular, χ^2^/ DF = 2.912 (<3), RESEA = 0.075 (<0.08), SRMR = 0.071 (<0.08), TLI (NNFI) = 0.917 (>0.9), CFI = 0.924 (>0.9), GFI = 0.956 (>0.9), AGFI = 0.920 (>0.9), indicating that the results of this study are acceptable.

**TABLE 5:** Model fit criteria and the test results.

Model fit	Criteria	Model fit of research model	Result
χ^2^	The small the better	818.15	
DF	The large the better	281	
Normed Chi-square (χ^2^/DF)	1 < χ^2^/DF < 3	2.912	Good
RESEA	<0.08	0.075	Excellent
SRMR	<0.08	0.071	Excellent
TLI (NNFI)	>0.9	0.917	Excellent
CFI	>0.9	0.924	Good
GFI	>0.9	0.956	Good
AGFI	>0.9	0.920	Good

### Regression Coefficient

In the model of this study (see Table 6), there is a significant effect of internal attributions on travel intention, that is, path coefficient = 0.358, *z*-value = 2.486, *p* < 0.05. There is a significant effect of external attribution on travel intention, that is, path coefficient = 0.330, *z*-value = 2.507, *p* < 0.05, and Hypothesis 1 is verified. There was a significant effect of internal attribution on affective empathy, that is, path coefficient = 0.615, *z*-value = 5.436, *p* < 0.05, and there was a significant effect of internal attributions on cognitive empathy, that is, path coefficient = 0.552, *z*-value = 5.719, *p* < 0.05. There was a significant effect of external attribution on affective empathy, i.e., path coefficient = 0.569, *z*-value = 5.478, *p* < 0.05. There was a significant effect of external attributions on cognitive empathy, that is, path coefficient = 0.274, *z*-value = 3.329, *p* < 0.05, and Hypothesis 2 was verified. There was a significant effect of affective empathy on travel intention, that is, path coefficient = 0.459, *z*-value = 2.226, *p* < 0.05. There was a significant effect of cognitive empathy on travel intention, that is, path coefficient = 0.140, *z*-value = 2.001, *p* < 0.05, and Hypothesis3 was verified.

**TABLE 6:** Regression coefficient.

DV	IV	Unstd	S.E.	*z*-value	*p*	Std.	R^2^	Result
AE	IA	0.443	0.082	5.436	***	0.615	0.702	Pass
	EA	0.383	0.070	5.478	***	0.569		Pass
CE	IA	0.568	0.099	5.719	***	0.552	0.379	Pass
	EA	0.263	0.079	3.329	***	0.274		Pass
TI	IA	0.276	0.111	2.486	*	0.358	0.990	Pass
	EA	0.240	0.096	2.507	**	0.333		Pass
	CE	0.105	0.053	2.001	*	0.140		Pass
	AE	0.491	0.221	2.226	**	0.459		Pass

*p < 0.05, **p < 0.01, ***p < 0.001.

*IA indicates sustained internal attribution, EA indicates external attribution, CE indicates cognitive empathy, AE indicates affective empathy, TI indicates travel intention.*

### Mediating Effect Analysis

In order to calculate the mediation effect more accurately, this study uses the confidence interval method (Bootstrap Distribution of Effects) (see Table 7) to analyze and test the mediation effect. The Bootstrap estimation technique is used to analyze the confidence intervals of the total effect, the indirect effect and the direct effect, and then the significance level of the mediation effect is further calculated (Hayes, 2009). The upper and lower limits of the bias-corrected 95% confidence interval do not contain “0,” which means the effect is passed. The total effect of internal attribution on travel intention was 0.553 with a *z*-value of 3.182, which met the criteria of >1.96. At 95% confidence level, the confidence interval obtained by bias-corrected estimation method is 0.422–0.726; the confidence interval obtained by percentile estimation method is 0.422–0.722, which does not contain 0, so the total effect holds. The indirect effect of internal attribution acting on travel intention through cognitive empathy was 0.060, and the *z*-value was 2.429, which met the criterion of >1.96. At 95% confidence level, the confidence interval obtained by bias-corrected estimation method is 0.010–0.163; the confidence interval obtained by percentile estimation method is 0.014–0.152, which does not contain 0. Therefore, the indirect effect of internal attribution acting on travel intention through cognitive empathy holds. The indirect effect of internal attribution acting on travel intention through affective empathy is 0.218, and the *z*-value is 2.036, which meets the criterion of >1.96. At 95% confidence level, the confidence interval obtained by bias-corrected estimation method is 0.011–0.781; the confidence interval obtained by percentile estimation method is 0.010–0.770, which does not contain 0. Therefore, the indirect effect of internal attribution acting on travel intention through affective empathy is holds.

**TABLE 7:** The analysis of mediation effect.

Effect	Point estimate	product of coefficients			Bootstrap			
					Bias-corrected 95%		Percentile 95%	
				
		S.E.	*z*-value	*p*-value	Lower	Upper	Lower	Upper
Total effect: IA → TI	0.553	0.174	3.182	*	0.422	0.726	0.422	0.722
Indirect effect: IA → CE → TI	0.060	0.025	2.429	*	0.010	0.163	0.014	0.152
Indirect effect: IA → AE → TI	0.218	0.107	2.036	*	0.011	0.781	0.010	0.770
Direct effect: IA → TI	0.276	0.128	2.150	*	0.256	0.554	0.275	0.550
Total effect: EA → TI	0.455	0.146	3.119	***	0.333	0.612	0.337	0.617
Indirect effect: EA → CE → TI	0.028	0.012	2.333	*	0.010	0.687	0.009	0.683
Indirect effect: EA → AE → TI	0.188	0.094	2.004	*	0.017	0.284	0.016	0.275
Direct effect: EA → TI	0.240	0.114	2.101	*	0.235	0.476	0.245	0.474

*p < 0.05, ***p < 0.001.

*IA indicates sustained internal attribution, EA indicates external attribution, CE indicates cognitive empathy, AE indicates affective empathy, TI indicates travel intention.*

The direct effect of internal attribution is 0.276 and the *z*-value is 2.150, which meets the criteria of >1.96. At 95% confidence level, the confidence interval obtained by bias-corrected estimation method is 0.256–0.554; the confidence interval obtained by percentile estimation method is 0.275–0.550, which does not contain 0. Therefore, the direct effect of internal attribution is valid. Therefore, hypothesis H4a holds and is a partial intermediary, and H4b is established as a partial intermediary. In the same way, H4c is established and is a partial intermediary, H4d is also established as a partial intermediary. In summary, it is clear that the empathy process (cognitive empathy and affective empathy) partially mediates the relationship between internal and external attributions (internal vs. external attributions) and travel intention.

## Research Discussion

### Conclusion

Based on a systematic review of meme theory, the planned behavior theory, and empathy theory, this paper tests the applicability of the behavior “Internet celebrity spots punch in” in the context of using online social software. Overall, the network user generated by three internal attribution requirements, accepted by the travel memes (meme content, meme form), and subjective norm are important pre-test influences on travel intention generated by travelers, which have a positive influence on travel intention, and their influence is mediated by the empathy process, namely, the empathy process mediates the relationship between internal and external attributions and travel intention. The details are as follows.

First, internal and external attribution have a significant positive effect on travel intention. The research results show that internal and external attribution have a significant positive impact on the willingness of the network user’s travel intention to Internet celebrity spots, which is in line with the meme diffusion theory proposed by Chesterman (2009) and similar to the research results of Mohammad et al. (2019). In the whole process of direct effect of symbolic consumption driving, the direct effect of internal attribution and external attribution on tourists’ intention is slightly different. The internal attribution is still slightly higher than the external attribution. With the continuous integration of media and tourism economy, the official tourism propaganda videos released by the government are quite different from those released by Internet celebrities and micro blog VIPs. Memes produced by Internet celebrities are good at using professional skills such as video clips to show the image jigsaw puzzle of destinations with short, fresh, and fast shots and scene switching, combined with background music and text description of the situation. The form of memes communication perfectly fits the reading habits of Internet users. It presents the personalized and rhythmic construction of meme elements such as destination landscape architecture and local cuisine. Meanwhile, with its strong fan community, a large number of tourists go there to “follow the trend to travel,” resulting in the “conformity effect.” Therefore, reasonable collocation and application of external attribution (memes content, memes form, subjective norms) is also an effective way to build web celebrity tourist destinations.

Second, internal and external attribution has a significant positive effect on empathy process. The results of this study are consistent with previous research conclusions, confirming the significant relationship between internal and external attribution and empathy process (Jang and Chung, 2019; Lénia and Beatriz, 2020). The results indicate that both internal attribution and external attribution have significant positive effects on cognitive empathy and emotional empathy, and internal attribution has a more significant effect on emotional empathy. In order to seek group approval, tourists will imagine others’ opinions about their behaviors before posting information about their status. Therefore, they often modify the published content repeatedly to obtain the recognition of the audience. Also, they will further strengthen or correct their cognition according to others’ likes or comments on themselves after releasing the information. All these behaviors will further enhance the cognitive empathy and affective empathy of tourists to the destination they punch in.

Third, the empathy process has a significant positive effect on travel intention. The results are consistent with those of Kim and Cooke (2021) and Yang et al. (2021b). Compared with cognitive empathy, the direct influence of affective empathy is more significant in the influence path from empathy to travel intention. The personalized psychological needs of consumers and the rich content production in the era of pan-entertainment constantly promote the transformation of “image fetishism.” Driven by the effect of web celebrity, the social capital and cultural capital produced by the new media social platform change the presence of tourists from the traditional physical space to virtual space. The most important thing in symbolic consumption is the emotional needs beyond the destination that tourists attach to the behavior of punching in, which further strengthen the generation of travel intention of users punching in.

Four, the process of empathy partially mediates the process of internal and external attribution and travel intention. The results are consistent with Eisenberg et al. (2015), which show that there are some mediations between the internal and external factors of empathy and behavior. The mediating effect of internal attribution on the intention to travel through affective empathy is the strongest, and the mediating effect of internal attribution on the intention to travel through cognitive empathy is the weakest. Internet users perceived affective empathy, namely individual tourists’ demand for the destination of the objective things and the corresponding experience emotional reaction, embodied in performance and subjective experience, external physical arousal, and compared with the cognitive empathy, the emotional response of affective empathy will prompt consumers to have stronger behavioral intention under the touch of internal attribution factors.

### Theoretical Implications

On the one hand, this study explores the internal and external theoretical dimensions of travel attribution of “Internet celebrity punch in” from the perspectives of meme theory and empathy theory, and explains the positive influence mechanisms of affective empathy and cognitive empathy on the travel intention. In recent years, on the basis of the development of classical tourism destination decision theory (planning behavior theory and tourism experience theory) (Ii and Gilmore, 1998; Grace and O’Cass, 2004; Wang et al., 2012), many scholars have begun to pay attention to behavioral variations and differences in media effects caused by tourism destination decision-making in the digital economy (Kowalczyk and Pounders, 2016; Giles, 2018). However, there is still little detailed research on attribution dimensions, especially quantitative research. This paper integrates various factors that influence the travel intention to Internet celebrity attraction and makes a systematic study by using quantitative analysis method, expanding the theoretical research of tourism marketing in the information age on the cross-disciplinary aspects of media platform psychological communication.

On the other hand, combining meme theory with tourist destination decision-making breaks through the traditional research paradigm of path research on tourism decision-making from the field of planned behavior. Meme theory combines with tourist destination decision to break the traditional research. In addition, there are few studies that combine the theory of empathy with the discipline knowledge of psychology. This paper explores the intrinsic psychological evolutionary processes that motivate and sustain individual activities and the attributional elements of behavioral motivation, relying on user psychological drive and emotional resonance motivation for tourism decision making, which not only enriches the literature on the theoretical foundations of empathic communication, net roots economy and tourism decision making but also provides a new way of thinking and perspective for other scholars to develop their research in the future.

### Practical Implications

First, it is clear that the internal and external factors that directly drive the generation of the intention to travel are important decisions to grasp the new situation of Internet tourism. Symbol consumption and web celebrity marketing have transformed the consumption pattern of tourism destinations from traditional landscape entertainment consumption to decentralized communication consumption of “multi-point interaction and collective performance.” This study focuses on two dimensions: internal attribution (self-cognition, self-presentation, group identity) and external attribution (meme content, meme form, subjective norm) and analyzes its influence mechanism on tourists’ decision to travel, which can help tourism destinations and relevant tourism enterprise departments to better grasp the communication subject and provide a basis for constructing the image discourse field of tourism destinations, so as to seize the opportunity to promote precise marketing of cultural symbols, promote products and services, improve tourism quality, and meet the needs of tourists.

Second, notice that the importance of a combination of internal and external attributive factors to trigger the consumers’ online empathy is the first step to promote the new tourism consumption. Developers and marketers of tourist destinations should make reasonable combination and comprehensive use of the significant effects of internal attribution and external attribution on affective empathy and cognitive empathy to arouse tourists’ resonance and emotional resonance. To keep costs under control, maximization of marketing benefits and feedback spillover can be achieved by adjusting the proportion of effects. In other words, at different stages of empathy transmission, when the dominant ratio of cognitive empathy and affective empathy changes dynamically, internal attributive stimuli can be used to strengthen tourists’ affective empathy, forming a comparative advantage, and maintaining the social heat of web celebrities to a certain extent to prolong the existence cycle of this media spectacle.

Third, the empathetic reaction before the emergence of user behavior provides a new idea for tourism destination marketing. In the process of empathy, affective empathy obviously has great situational, exciting, and transient characteristics, and the emotions of some tourists of web celebrity attractions can inadvertently spread to others. When tourists are in a new and unfamiliar environment, their emotional experience is generally strong. If individuals are affected by negative emotional experience, they are prone to cognitive bias, which leads to their behaviors being controlled by emotions, resulting in adverse consequences. Therefore, it is particularly important to regulate the overall Internet emotional atmosphere of web celebrity attractions marketing in the influence path of empathy process on the intention to travel.

Four, as a key link, it is necessary to study the mediating effect of empathy communication on tourists’ intention to travel. The birth of travel to web celebrity attractions consumption mode caters to the media practice development trend of square carnival and visual consumption under the Internet celebrity economy. Starting from the visual consumption scene planned by merchants, “web celebrity attractions” follows the communication and circulation of virtual network as well as the consumption, re-communication, and reproduction of netizens, thus forming a complete industrial chain of multi-point interest resonance and a media spectacle of multi-force collusion. Analysis platform for the Internet media, therefore, producing bidirectional empathy experience of consumers tend to attach great importance to travel. The reason is that travel ceremony can bring positive psychological cues, so the tourist attractions can be set up, such as love lock, cliff, coin-operated pool, time email, and everything that brings a positive attitude to visitors.

### Limitations

There are still many shortcomings in this study to be explored and improved in follow-up. For example, the data collected are point-in-time data from a certain time period on social media platforms, and the emotional changes of visitors may be heterogeneous depending on the stage of empathy transmission. For example, although the study discussed the distinction between internal attribution and external attribution in terms of the degree of influence of empathy, it did not consider the individual heterogeneity of tourists, namely, whether differences in personal life patterns have a significant impact on travel intention. However, due to the validity of the data, we did not consider whether individual heterogeneity, that is, differences in personal lifestyles, had a significant impact on travel intention. Therefore, there is a strong need for future research to obtain a more high-quality and extensive data set to further test the moderating effect of lifestyle.

## Data Availability Statement

The raw data supporting the conclusions of this article will be made available by the authors, without undue reservation.

## Author Contributions

KY and QW: conceptualization and formal analysis. KY, JX, and BL: data curation. KY and JX: investigation. KY, QW, and JX: writing original draft. KY, QW, and BL: writing–review and editing. All authors have read and agreed to the published version of the manuscript.

## Conflict of Interest

The authors declare that the research was conducted in the absence of any commercial or financial relationships that could be construed as a potential conflict of interest.

## Publisher’s Note

All claims expressed in this article are solely those of the authors and do not necessarily represent those of their affiliated organizations, or those of the publisher, the editors and the reviewers. Any product that may be evaluated in this article, or claim that may be made by its manufacturer, is not guaranteed or endorsed by the publisher.
